# Determining the maximum lanthanum incorporation in the fluorite structure of La-doped ceria nanocubes for enhanced redox ability†

**DOI:** 10.1039/c8ra09766f

**Published:** 2019-02-26

**Authors:** Danilo Loche, Lucy M. Morgan, Alberto Casu, Gavin Mountjoy, Colm O'Regan, Anna Corrias, Andrea Falqui

**Affiliations:** School of Physical Sciences, University of Kent Ingram Building Canterbury CT2 7NH UK a.corrias@kent.ac.uk; Biological and Environmental Sciences and Engineering Division, King Abdullah University of Science and Technology 23955-6900 Thuwal Kingdom of Saudi Arabia a.falqui@kaust.edu.sa

## Abstract

Ceria nanocubes have been doped with increasing amounts of lanthanum to enhance their redox ability. X-ray diffraction and transmission electron microscopy techniques provide a consistent picture indicating that there is an upper limit to the lanthanum that can be incorporated in the fluorite structure of ceria nanocubes, which is close to 7.5 mol% La. This limited loading is nevertheless able to produce a significant enhancement of the ceria redox ability as evidenced by use of X-ray absorption spectroscopy to determine the Ce^3+^/Ce^4+^ ratio in samples submitted to a degassing treatment at room temperature.

## Abstract 2

Ceria nanocubes have been doped with increasing amounts of lanthanum to enhance their redox ability.

## Graphical abstract

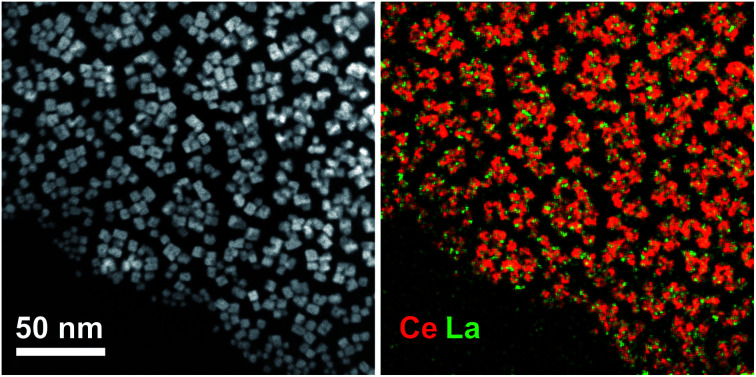

## Introduction

Ceria nanomaterials have attracted much attention due to their extraordinary reactivity, stemming from the capacity to store and release oxygen ions depending on the oxygen partial pressure of the environment.^1–6^ This reactivity originates from the labile and reversible redox cycle between Ce^4+^ and Ce^3+^, which is enhanced at the nanoscale and generates the equilibrium given in eqn (1), using the Kröger–Vink notation:1
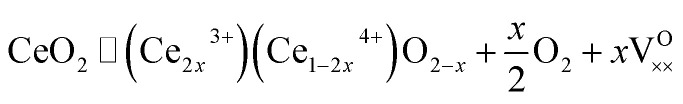


This reaction indicates that the ability of ceria to intake oxygen and release it (oxygen storage capacity) is driven by the vacancies that can be accommodated within the CeO_2_ fluorite structure. The oxygen storage capacity can be enhanced by adjusting the nanoparticle size and shape, and/or through the use of dopants.^7^

Thermal techniques, including hydrothermal and solvothermal synthesis,^8–10^ combustion/decomposition,^11,12^ and spray pyrolysis,^13^ among other techniques, have been used for the synthesis of ceria nanoparticles, producing nanoparticles of cuboidal,^14^ spherical,^15^ polyhedral,^16^ and truncated octahedron shapes.^3^

Cuboidal ceria nanoparticles are terminated predominantly by highly reactive 〈100〉 facets. This morphology provides the largest benefits in term of reactivity, as opposed to truncated octahedron nanoparticles which are predominantly terminated by 〈111〉 highly stable facets. It has also been shown that incorporating La^3+^ into the ceria lattice can create defects, including oxygen vacancies, due to the difference in the ionic radius between Ce^4+^ (0.097 nm) and La^3+^ (0.110 nm),^17^ which should further increase reactivity. There are studies which show that the cubic fluorite structure of ceria is maintained with the incorporation of lanthanum,^18^ however the extent of doping that can be achieved in highly reactive nanocubes has not been established.

In this paper we address, for the first time, the achievable limit of lanthanum doping in cuboidal ceria nanoparticles. We also study in detail the distribution of lanthanum within the nanocubes, and provide evidence of the stabilisation of oxygen vacancies due to the presence of lanthanum. We address these issues *via* a combination of powder X-Ray Diffraction (XRD), Conventional Transmission Electron Microscopy (CTEM), Spherical Aberration-Corrected High Resolution Transmission Electron Microscopy (AC-HRTEM), Spherical Aberration-Corrected Scanning Transmission Electron Microscopy – Energy Dispersive X-ray Spectroscopy (AC-STEM-EDS), and X-ray Absorption Near Edge Structure (XANES) at the Ce L_3_ edge. The combination of these techniques is of paramount importance since XRD is able to determine accurately the effect of La doping on the fluorite lattice constant, advanced electron microscopy techniques are able to study in detail the morphology, structure, and composition of the cuboidal nanoparticles, and XANES is able to determine the Ce^3+^/Ce^4+^ ratio which is a measure of the oxygen storage capacity.

## Experimental

### Synthesis of nanocubes

All materials used were of analytical purity or higher. The synthesis process followed is similar to that of Yang *et al.*^14^ To obtain CeO_2_ nanocubes, 15 mL of a 16.7 mmol L^−1^ water solution of Ce(NO_3_)_3_·6H_2_O (99.99%, Sigma-Aldrich) was placed in a Teflon-lined, stainless-steel autoclave (45 mL, Parr). To this solution 15 mL toluene (HPLC grade, Fisher), 1.5 mL oleic acid (extra pure, SLR, Fisher), and 0.15 mL *tert*-butylamine (98%, Sigma-Aldrich) were added. The autoclave was sealed tightly and transferred to a temperature-controlled electric oven where it was subjected to a hydro-solvothermal treatment at 180 °C for 48 h, before cooling to room temperature. The organic layer was then separated and purified by centrifugation to eliminate impurities. 30 mL of absolute ethanol (analytical grade, Fisher) was added to the purified solution to promote precipitation of the nanocubes. The solid precipitate was removed and dried at room temperature under air overnight. For doped nanocubes, La(NO_3_)_3_·6H_2_O (99.99%, Sigma-Aldrich) was mixed in the relative concentration with the Ce(NO_3_)_3_·6H_2_O precursor in aqueous solution, in order to achieve La-doping of 2.5, 5, 7.5, 10 and 12.5 mol%. The same synthesis process as for the ceria nanocubes was then followed.

### Characterisation

Powder X-ray Diffraction Patterns (XRD) were recorded on a PANalytical X'Pert3 diffractometer equipped with an X'Celerator linear detector and Cu Kα line source, scanning a 2*θ* range of 10–90°. XRD samples were prepared by placing one drop of a toluene solution of the nanocubes onto a zero-background sample holder and left to dry. The average size of crystallite domains was calculated using the Scherrer equation corrected for instrumental broadening using a LaB_6_ standard.

Both conventional (CTEM) and spherical aberration (C_S_)-corrected high resolution transmission electron microscopy (AC-HRTEM) were used to characterize the morphological and structural features of the different materials. C_S_-corrected HRTEM was performed by using a FEI Titan Cube microscope corrected for spherical aberration of the S-Twin objective lens by means of a CEOS corrector, working at an acceleration voltage of 300 kV, equipped with an ultra bright field emission electron source (X-FEG) and a Gatan 2k × 2k CCD camera. Energy Dispersive X-Ray Spectroscopy (EDS) compositional maps were performed in C_S_-corrected Scanning TEM (AC-STEM) mode in High Angle Angular Dark Field geometry, by using a double C_S_-corrected Thermoscientific Titan Themis Cube Microscope, working at an acceleration voltage of 300 kV and equipped with X-Twin objective lens, an ultra bright field emission electron source (X-FEG), a Ceta 4k × 4k CMOS camera and a Super-X EDS detection system, constituted by four independent windowless SDD EDS detectors, each with a detection surface area of 30 mm^2^, for an ultimate solid collection angle of 0.9 sr. The quantification of La mol% in La-doped CeO_2_ nanoparticles was performed, collecting the whole EDS spectra corresponding to the acquired maps, then calculated by using the Cliff–Lorimer method on the Lα peaks of both La and Ce. The peaks were fitted by using a Gaussian profile, after careful background subtraction. Errors in the corresponding quantifications are intended on the last significant digit.

XANES spectra at the Ce L_3_-edge were recorded on the B18 beamline at the DIAMOND synchrotron (Oxfordshire, UK), at room temperature in transmission mode using a Si (311) monochromator on the undoped CeO_2_ nanocubes, and the 7.5 mol% La-doped CeO_2_ nanocubes, both before and after a degassing treatment under vacuum (10^−6^ torr for 15 hours) at room temperature. Data were also collected on Ce^4+^ and Ce^3+^ reference compounds (CeO_2_, 99.9% Aldrich, and Ce(NO)_3_·6H_2_O, 99.99% Aldrich). The energy scale of the monochromator was calibrated *via* a third ion chamber using a Cr reference foil. Samples were mixed with polyvinylpyrrolidone (PVP) and pressed to form pellets. For the samples submitted to degassing under vacuum these operations were carried out inside an argon filled glove box, where the pellets were also sealed inside an aluminium pouch, to avoid any contact with air. The data analysis was performed using the ATHENA software.^19^ The linear combination fitting tool in ATHENA was used to determine the relative amounts between Ce^3+^ and Ce^4+^.

## Results and discussion

In Fig. S1† a typical TGA/DSC trace obtained under air flow for all the investigated samples is shown. A significant weight loss is observed in the TGA at around 400 °C, accompanied by an exothermic peak in the DSC. This is due to the oleic acid capping the nanocubes, which is burned off at this temperature. No significant differences were detected as a function of La-doping.

**Fig. 1:**
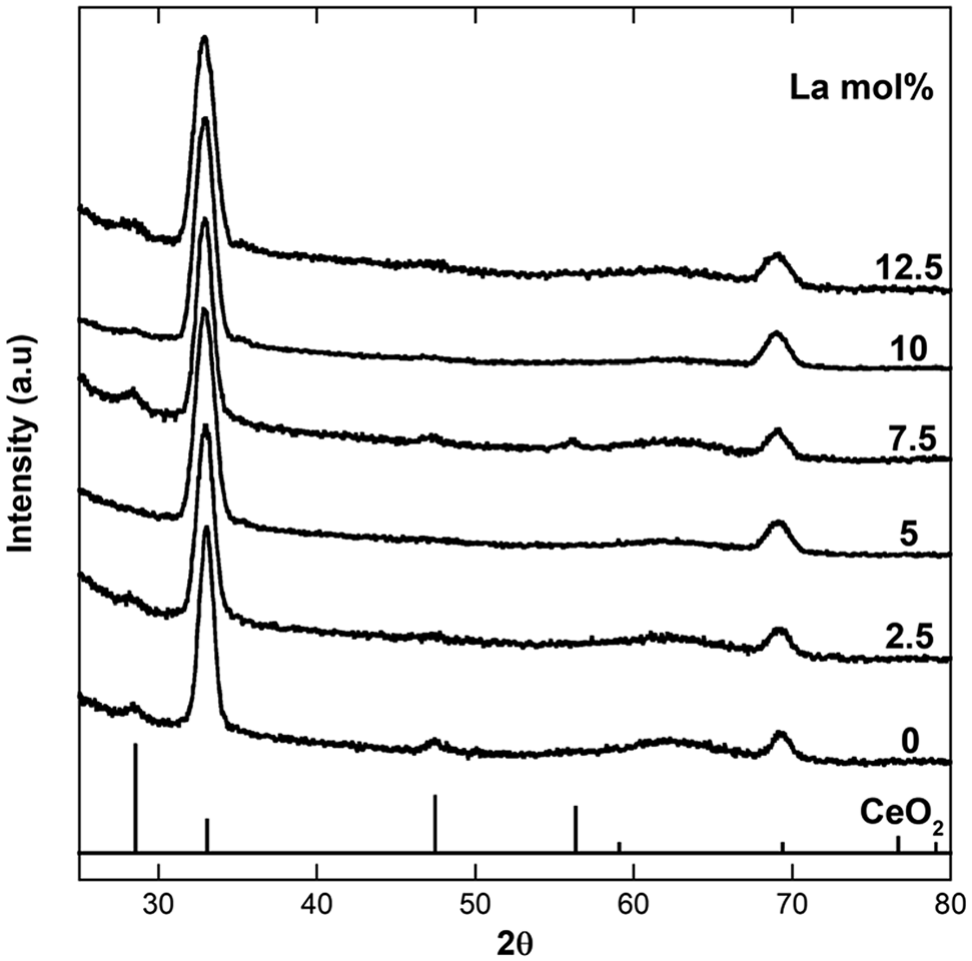
XRD patterns of undoped ceria nanocubes and ceria nanocubes with increasing La-doping.

Fig. 1 shows the XRD patterns of the samples with increasing La-doping compared to undoped CeO_2_ nanocubes. Due to the presence of the surfactant the nanocrystals self-assemble onto the sample holder following slow solvent evaporation from the toluene solution, with a similar arrangement as in the TEM images shown in the following. This leads to preferential orientation, and in fact all the patterns display relative peak intensities typical of self-assembled nanoparticles with a cubic shape, where the {200} and {400} peaks of the ceria fcc fluorite structure are the most prominent. This is because they are the only planes which are in the condition to diffract, compared to the almost undetectable {111} and {220} peaks, which are not in the correct orientation to provide detectable diffraction. Fig. S2† shows the typical pattern obtained depositing the samples on the holder as dried powders. In this case, the relative peak intensities are those typical of ceria with no preferential orientation effects, since no self-assembly takes place. The fluorite structure is retained for all the investigated samples, and no sign of additional phases and/or impurities is present.

**Fig. 2:**
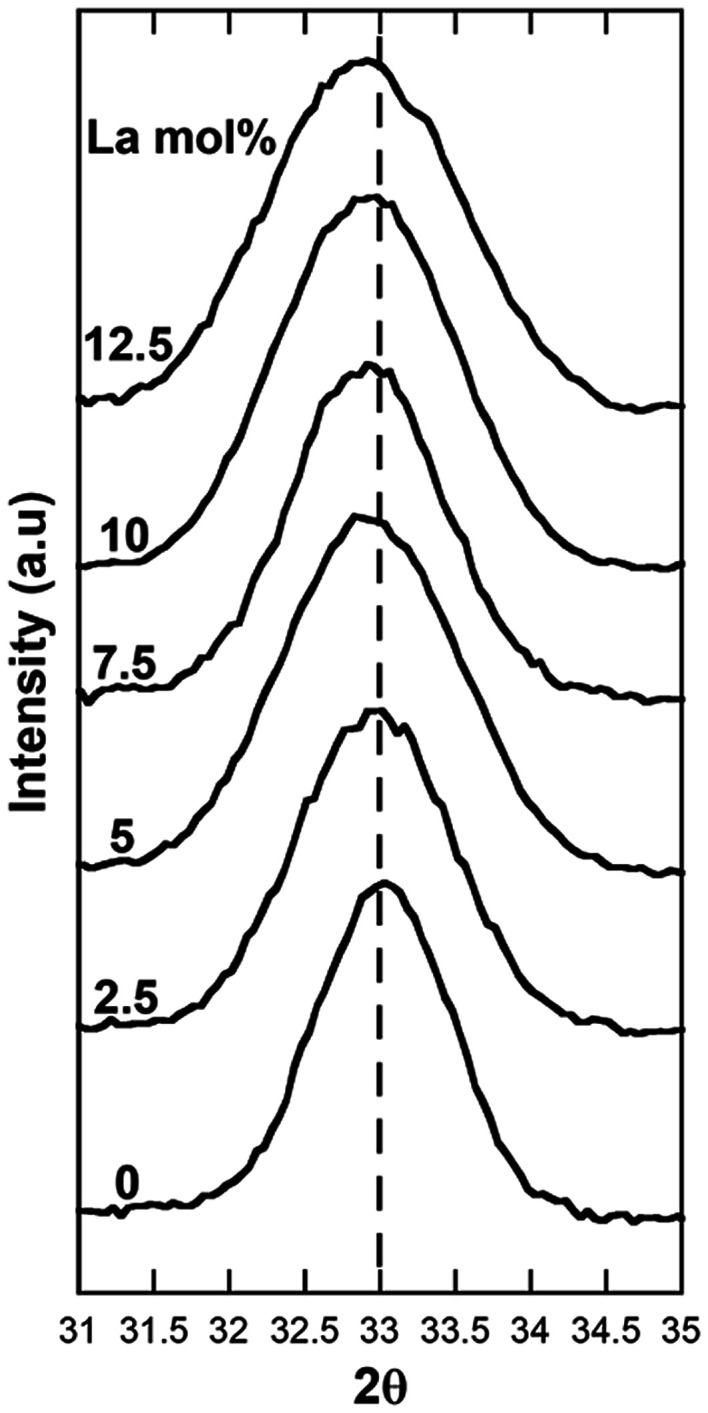
Detail of the Bragg {200} peak region for undoped ceria nanocubes and ceria nanocubes with increasing La-doping.

In Fig. 2 the region of the {200} Bragg peak is shown in detail. The values of the peak position, obtained by fitting the peak profile, and the corresponding lattice parameter are reported in Table 1, together with the crystallite sizes, calculated using Scherrer's formula, taking into account the instrumental broadening.

**Table 1:** Nominal La-doping (mol%), La-doping as determined by STEM-EDS, {200} Bragg peak position (2*θ*°), lattice parameter (Å) and crystallite size (nm) for the undoped and La-doped ceria nanocubes

La (mol%)	La STEM-EDS (mol%) [error ±0.5]	{200} peak position (2*θ*°) [error ±0.005]	Lattice parameter (Å) [error ±0.001]	Crystallite size^a^ (nm)
0	—	32.994	5.425	8.4
2.5	—	32.936	5.435	7.1
5.0	4.4	32.915	5.438	6.3 (5.9)
7.5	5.5	32.887	5.443	7.4 (5.0)
10	6.7	32.886	5.443	6.5 (6.5)
12.5	—	32.877	5.444	5.9

a Calculated from the broadening of the {200} peak. In bracket the average size estimated from the TEM images is reported.

The values of the lattice parameter obtained for all the samples are higher compared to that of bulk ceria (5.415 Å), indicating the presence of some Ce^3+^ and oxygen vacancies in the crystalline structure, even for the undoped ceria nanocubes.^20^ Moreover, a slight progressive shift of the {200} Bragg peak to lower angles, and corresponding increase of the lattice parameter, is clearly detectable with increasing La-doping up to 7.5 mol%, while the position of the peak does not shift further for higher loadings.

**Fig. 3:**
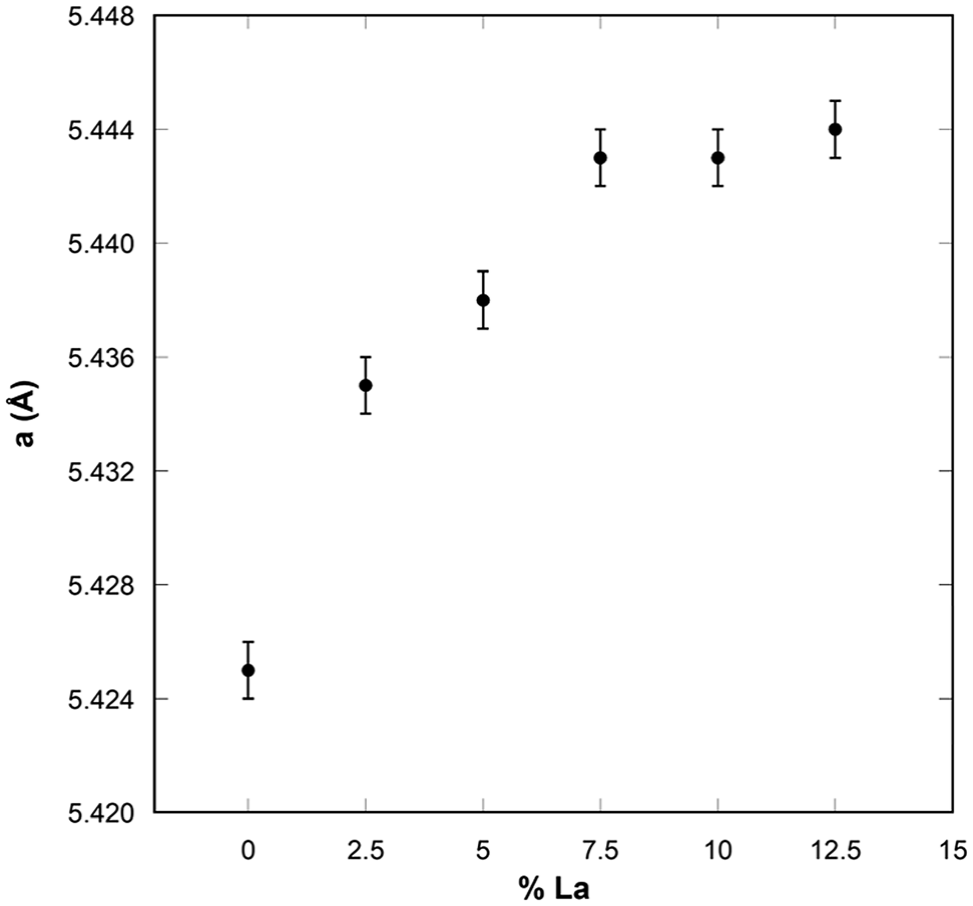
Lattice parameter of undoped and La-doped ceria nanocubes.

Further insight can be derived from the inspection of Fig. 3 showing the lattice parameter as a function of the La-doping, where again it can be noticed that the lattice parameter increases progressively up to 7.5 mol% of nominal La-doping and then tends to a plateau for higher loadings. These results indicate that the Vegard's law is only followed up to 7.5 mol% La-doping, with this loading corresponding to the upper limit of La that can be accommodated in the fluorite crystalline structure of ceria forming a solid solution. As La^3+^ is progressively incorporated, an expansion of the lattice is observed due to the slightly larger La^3+^ ionic radius (116 pm) compared to that of Ce^4+^ (97 pm). It should be noted that despite the similar ionic radii of Ce^4+^ and La^3+^, which in principle would allow solid solubility within all compositions in mixed Ce/La oxides, the structure of the two parent oxides is remarkably different, with the stable La_2_O_3_ crystalline phase being hexagonal. Moreover due to the different oxidation states between Ce^4+^ and La^3+^, for the latter to be incorporated within the fluorite structure vacancies need to be accommodated. A much wider range of solid solubility compared to the range observed in this study has been reported by Morris *et al.*,^21^ on mixed Ce/La oxides prepared by conventional precipitation and also for mesoporous nanostructured mixed Ce/La oxides.^18^ However, the authors of the latter study could not explain why the mixed oxide phases favored the fluorite crystal structure at La contents as high as 90% and the presence of an amorphous component in their samples could not be ruled out.

Despite the wide number of studies regarding the doping of ceria nanoparticles, only a few are focused on the doping of ceria nanocubes. A wide concentration range has not been explored to date in La/Ce oxide nanocubes to determine whether there is a limit in the doping that can be achieved while retaining the fluorite structure. Fernandez *et al.*^22^ in their study on 10 mol% La-doped ceria nanocubes reported a peak shift in their XRD patterns, with the lattice parameter changing from 5.42 Å for pure ceria nanocubes to 5.44 Å for 10% La-doped CeO_2_. This value is consistent with our findings (Table 1). Our work, expanding the study to a large range of La doping levels, points out that once the upper limit is reached, no further lanthanum can be incorporated regardless of the additional amount of La^3+^ used in the synthetic step. Similar findings have been reported by Mendiuk *et al.* on the limit of doping that can be achieved in ceria nanocubes with other lanthanides, in particular, 10 mol% for Pr, 20 mol% for Gd and Sm, and 30 mol% for Tb and Er.^23,24^ It should be noted that in ref. 23 and 24 the increase in the amount of dopant induces a loss of the cubic shape as well as a modification of the nanocubes sizes. A mixture of nanocubes and nanorods has also been obtained in Praseodymium doped CeO_2_ nanocubes recently investigated by L. Jiang *et al.*,^25^ for Pr doping higher than 15%. It should be noted that the hydrothermal method used in ref. 25 does not include the use of a capping agent, leading to nanocubes which are much larger than the ones obtained using the synthetic approach of this study.

Fig. 2 and Table 1 also show an increase in the peak broadening for all the samples containing La^3+^ compared to that of the undoped CeO_2_ sample, indicating that La-doping produces a decrease of crystallite domains size. This downsizing effect upon doping has been previously observed by Bezkrovnyi *et al.* on Eu-doped ceria nanocubes,^26^ but is in contrast with respect to what reported by Fernandez *et al.* for La-doped ceria nanocubes.^22^ It should be pointed out that the formation of vacancies produced by La-doping might also be contributing to the peak broadening.

**Fig. 4:**
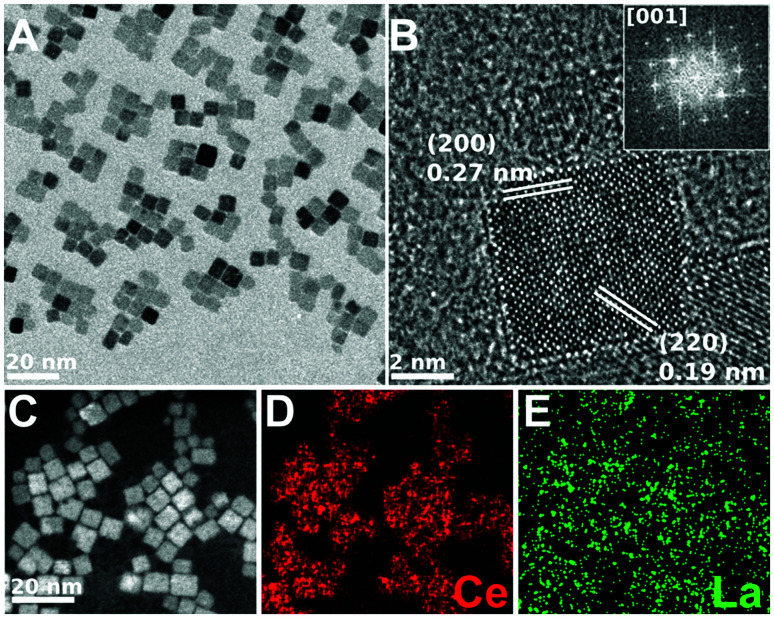
CTEM, AC-HRTEM, HAADF-AC-STEM, and AC-STEM-EDS mapping of the nominal 5 mol% La-doped CeO_2_ nanocubes. (A) CTEM image showing clusters of La-doped CeO_2_ nanocubes. (B) AC-HRTEM image showing a single crystalline La-doped CeO_2_ nanocube projected along the [001] zone axis, with the *d*-spacings for the (200) and (220) lattice planes being shown. Inset shows the corresponding 2D-FFT. (C) HAADF-AC-STEM image of the same nanocubes, showing the scan region imaged for EDS mapping. (D and E) EDS maps showing the distribution of Ce and La throughout the nanocubes, respectively. The EDS overall quantification indicates a molar percentage of Ce and La of 95.6% and 4.4%, respectively.

**Fig. 5:**
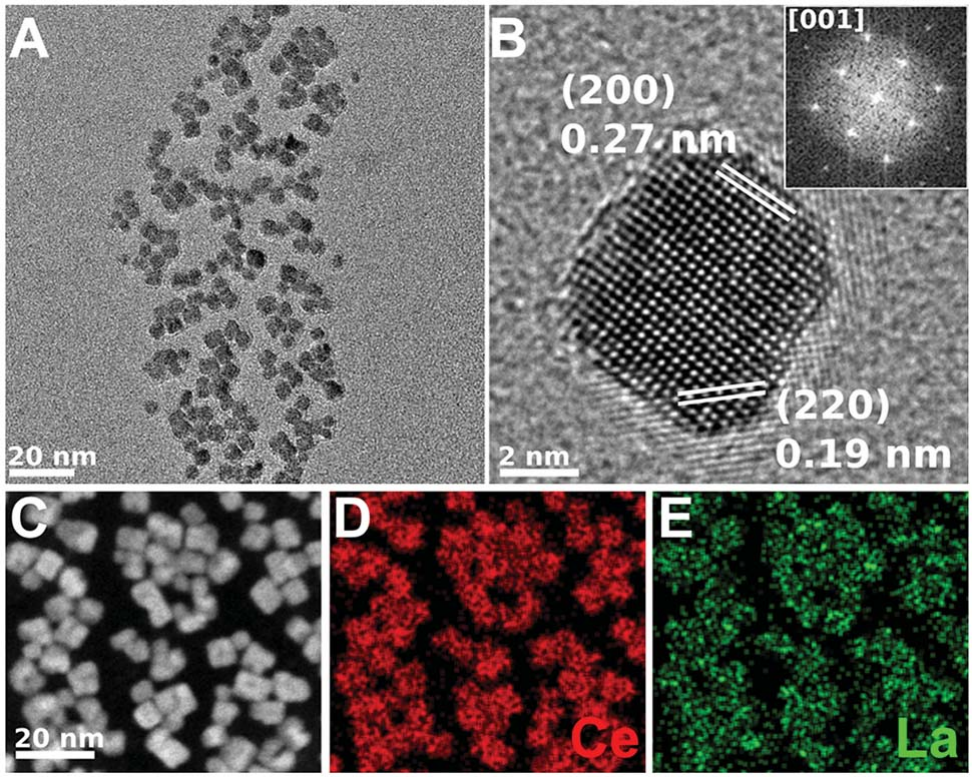
CTEM, AC-HRTEM, HAADF-AC-STEM, and AC-STEM-EDS mapping of the nominal 7.5 mol% La-doped CeO_2_ nanocubes. (A) CTEM image showing clusters of La-doped CeO_2_ nanocubes. (B) AC-HRTEM image showing a single crystalline La-doped CeO_2_ nanocube projected along the [001] zone axis, with the *d*-spacings for the (200) and (220) lattice planes being shown. Inset shows the corresponding 2D-FFT. (C) HAADF-AC-STEM image of the same nanocubes, showing the scan region imaged for EDS mapping. (D and E) EDS maps showing the distribution of Ce and La throughout the nanocubes, respectively. The EDS overall quantification indicates a molar percentage of Ce and La of 94.5% and 5.5%, respectively.

**Fig. 6:**
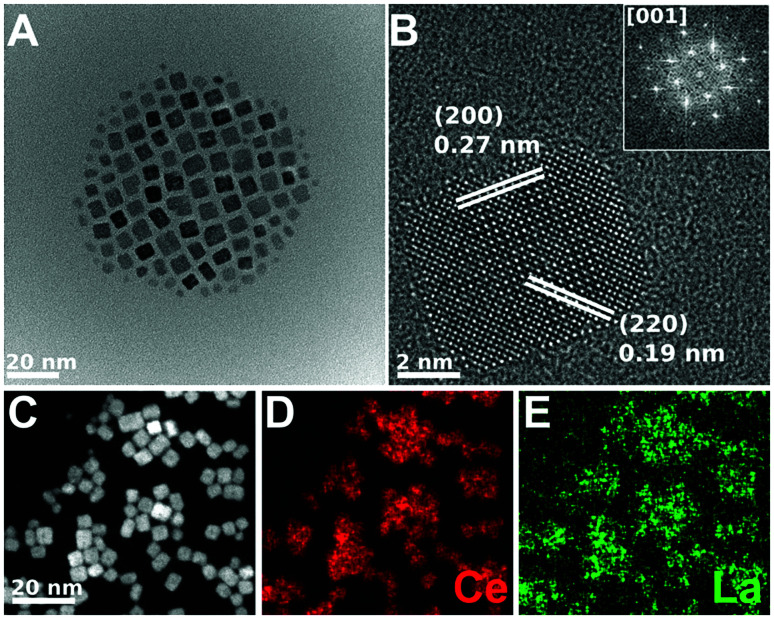
CTEM, AC-HRTEM, HAADF-AC-STEM, and AC-STEM-EDS mapping of the nominal 10 mol% La-doped CeO_2_ nanocubes. (A) CTEM image showing clusters of La-doped CeO_2_ nanocubes. (B) AC-HRTEM image showing a single crystalline La-doped CeO_2_ nanocube projected along the [001] zone axis, with the *d*-spacings for the (200) and (220) lattice planes being shown. Inset shows the corresponding 2D-FFT. (C) HAADF-AC-STEM image of the same nanocubes, showing the scan region imaged for EDS mapping. (D and E) EDS maps showing the distribution of Ce and La throughout the nanocubes, respectively. The EDS overall quantification indicates a molar percentage of Ce and La of 93.3% and 6.7%, respectively.

Fig. 4–6 show the CTEM, AC-HRTEM, and AC-STEM-EDS maps of three La-doped CeO_2_ nanocubes samples, with 5 mol%, 7.5 mol%, and 10 mol% nominal La-doping. Fig. S3† shows the EDS spectra corresponding to the maps presented in Fig. 4–6.

CTEM shows that in all the samples the cubic shape is well maintained and that each nanocube is mono-crystalline with the sides corresponding to the six {001} crystalline facets. Moreover, the *d*-spacing obtained from AC-HRTEM is in agreement with the value expected for CeO_2_. Considering AC-HRTEM point resolution is 0.8 Å, the variation of the lattice parameter induced by La-doping is too subtle to be detectable in the AC-HRTEM images.

All the AC-STEM-EDS compositional maps show that La is uniformly distributed throughout the nanocubes. The amounts of La determined by AC-STEM-EDS for the samples with 5 and 7.5 mol% nominal La-doping are slightly lower than the nominal value, but within the experimental error (see Table 1). Instead, for the sample with 10 mol% nominal La-doping the amount determined by AC-STEM-EDS is significantly lower, 6.7 mol%, and quite close to the upper limit of La-doping that can be incorporated into the fluorite structure of ceria, as determined by XRD analysis. The compositional analysis therefore confirms that if the amount of La^3+^ used in the synthetic step is higher than the upper limit, the excess will not react and a higher loading cannot be achieved.

All the results are consistent with the synthetic approach used in our study leading to homogeneous La-doping within the nanocubes, no additional phases being formed for any explored composition, even if there is a maximum La amount that can be incorporated in the nanocubes. Compared to other synthetic approaches that might lead to a wider range of solid solubility, the used approach offers the advantage of being shape and size selective, with nanocubes smaller than 10 nm, and with a narrow distribution of sizes. This is achieved thanks to the use of oleic acid, which mediates the growth at the very early stages of the hydrothermal synthesis. Surfactant mediated growth has the disadvantage of producing small amounts of nanocubes, but could be scaled up by the use of larger autoclaves.

Moreover, due to the controlled shape and size our nanocubes are expected to be highly reactive, *via* a stabilisation of oxygen vacancies. This was confirmed by performing XANES experiments to probe the Ce^3+^/Ce^4+^ ratios as a function of composition and pre-treatment of the nanocubes.

**Fig. 7:**
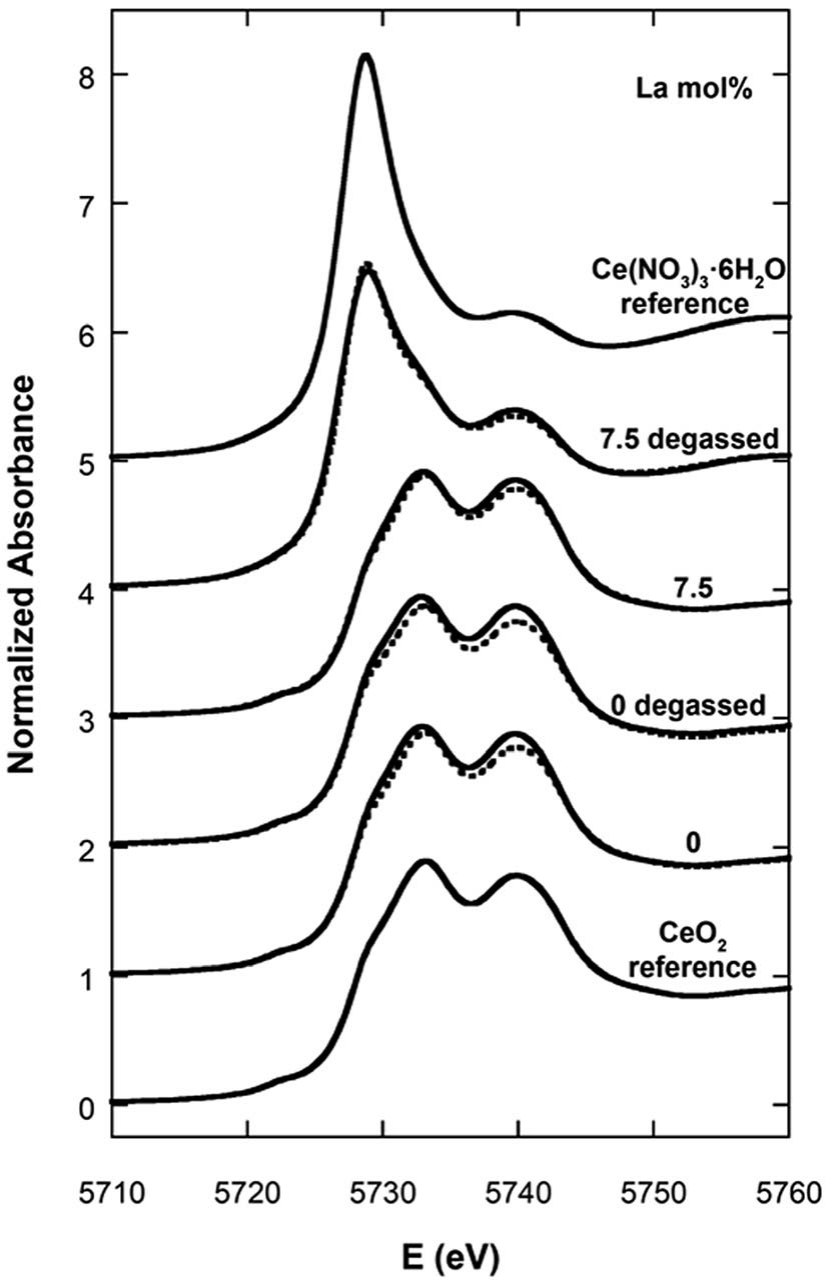
XANES spectra for the undoped and 7.5 mol% La-doped CeO_2_ nanocubes before and after degassing treatment, compared with Ce^3+^ and Ce^4+^ reference compounds. The calculated spectra obtained by linear combination fitting analysis are shown as dashed lines.

Fig. 7 shows the XANES spectra for the undoped CeO_2_ nanocubes and for the 7.5 mol% La-doped CeO_2_ nanocubes (*e.g.* the sample with the highest achievable loading), both before and after a degassing treatment under vacuum at room temperature. In the same figure the XANES spectra of the CeO_2_ and Ce(NO)_3_·6H_2_O reference compounds are also shown. The two samples before degassing are very similar to the CeO_2_ reference compound. Instead, the effect of the degassing is remarkably different between the undoped and the La-doped sample. In particular, for the latter sample degassing is accompanied by a notable increase in the amount of Ce^3+^. In fact, the spectrum of the La-doped sample after degassing is much more similar to the Ce^3+^ reference compound indicating that lanthanum doping has a very strong stabilisation effect on Ce^3+^ and therefore on the oxygen storage capacity. The relative fractions of Ce^3+^ and Ce^4+^ determined by linear combination fitting analysis are reported in Table 2.

**Table 2:** Relative amounts of Ce^3+^ and Ce^4+^ for undoped and 7.5 mol% La-doped CeO_2_ nanocubes before and after degassing treatment, as determined by linear combination fitting of CeO_2_ and Ce(NO)_3_·6H_2_O reference spectra

La (mol%) and treatment	% Ce^3+^	% Ce^4+^
Undoped	1.1 ± 0.5	98.9 ± 0.7
Undoped degassed	4.9 ± 0.5	95.1 ± 0.5
7.5	0.0 ± 0.3	100.0 ± 0.5
7.5 degassed	68.4 ± 0.3	31.6 ± 0.6

In the two samples before degassing almost all cerium is present as Ce^4+^. Once submitted to the degassing treatment just 5% of the cerium is reduced to Ce^3+^ in the undoped sample, while almost 70% is reduced to Ce^3+^ in the sample with the highest achievable La-doping of 7.5 mol%. These results obtained *via* a degassing treatment at room temperature demonstrate that with just 7.5% mol of La-doping a large fraction of vacancies can be effectively generated, with a great beneficial effect on the oxygen storage capacity.

It should be noted that reducibility and oxygen storage capacity of La and Pr doped ceria nanocubes has been previously studied in ref. 22 and 25 using temperature programmed reduction experiments. In the above mentioned papers, reduction is not significant up to temperatures around 450 °C, with the most important events taking place in the 500–700 °C range, and XPS shows a very limited amount of Ce^3+^.

Since XANES probes the Ce oxidation state of the whole sample while XPS probes the oxidation state within a few atomic layers from the surface, our results point out that by controlling the size and shape of La-doped nanoparticles, the stabilisation of oxygen vacancies can be greatly enhanced.

## Conclusions

The synthesis of La-doped CeO_2_ nanocubes has been approached with a wide range of La-doping. Our findings prove that the maximum amount of lanthanum that can be incorporated while maintaining the ceria fluorite structure is close to 7.5 mol%, using the surfactant mediated growth approach used in this study. When a larger La/Ce ratio is used in the synthesis the compositional analysis points out that the lanthanum present in the nanocubes is close to this upper limit and the additional lanthanum does not react during the hydro-solvo-thermal synthesis. Lanthanum is homogenously distributed within the nanocubes for all samples. The nanocubes with the largest achievable La-doping show a remarkable oxygen storage capacity due to the combined effect of the stabilisation of the oxygen vacancies induced by the doping and the control of both the shape and size of the nanoparticles terminated by highly reactive 〈100〉 facets. Such nanocubes are expected to have optimized catalytic performance in several reactions such as water-gas-shift.

## Conflicts of interest

There are no conflicts to declare.

## Supplementary Material

RA-009-C8RA09766F-s001
