# Supplementary material for: Determining the maximum lanthanum incorporation in the fluorite structure of La-doped ceria nanocubes for enhanced redox ability
Source: RSC Adv. 2019 Feb 26;9(12):6745–51. doi: 10.1039/c8ra09766f (PMC9061073; doi:10.1039/c8ra09766f)
Supplement: RA-009-C8RA09766F-s001 [file RA-009-C8RA09766F-s001.pdf]

Electronic Supplementary Material (ESI) for RSC Advances.
This journal is © The Royal Society of Chemistry 2019

Electronic Supplementary Information

Determining the maximum lanthanum incorporation in the fluorite structure of La-doped ceria nanocubes for enhanced redox ability

Danilo Loche,[a] Lucy M Morgan,[a] Colm O'Regan,[b] Gavin Mountjoy,[a] Alberto Casu,[b] Anna Corrias,*[a] and Andrea Falqui*[b]

[a] School of Physical Sciences, University of Kent, Ingram Building, Canterbury, CT2 7NH, UK.

[b] Biological and Environmental Sciences and Engineering Division King Abdullah University of Science and Technology, 23955-6900 Thuwal, Kingdom of Saudi Arabia.

Thermal Gravimetric Analysis (TGA) and Differential Scanning Calorimetry (DSC)

TGA and DSC measurements were carried out on the dried samples using a Netzsch STA 409 PC, heating the samples from 25 °C to 600 °C at 10 °C/min, under a flow of air.

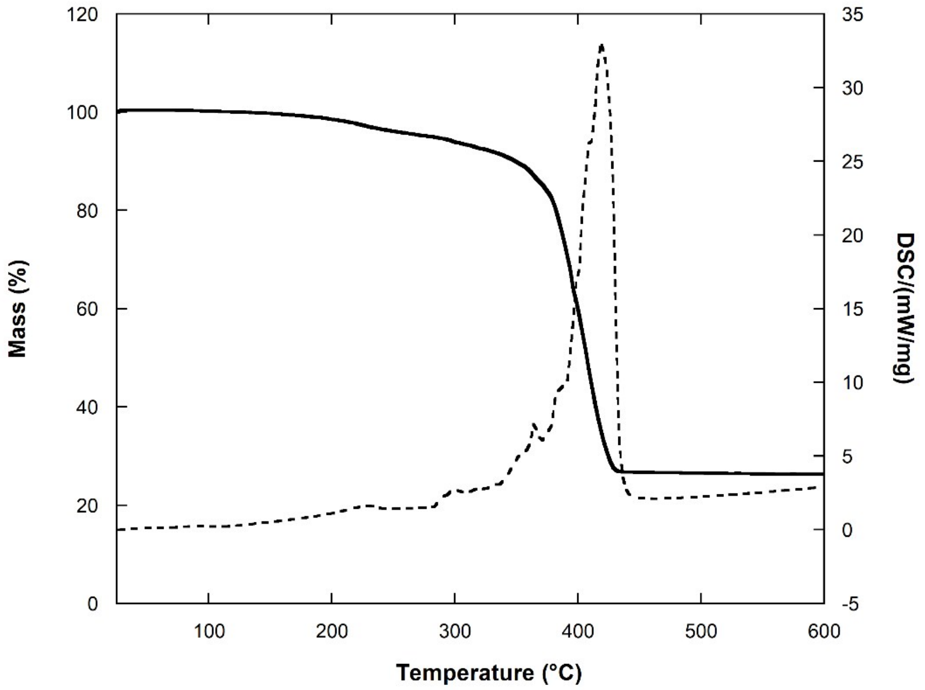

Fig. S1 TGA (continuous line) and DSC (dashed line) traces for undoped CeO_2 nanocubes.

Figure S1 shows the typical TGA and DSC traces that were obtained for all samples. A large amount, close to 80 wt%, of oleic acid is capping the particles which starts to decompose at temperatures above 200 °C. The main weight loss is close to 400 °C and is accompanied by a strong exothermic peak in the DSC due to the complete combustion of the oleic acid.

Powder X-ray Diffraction (XRD)

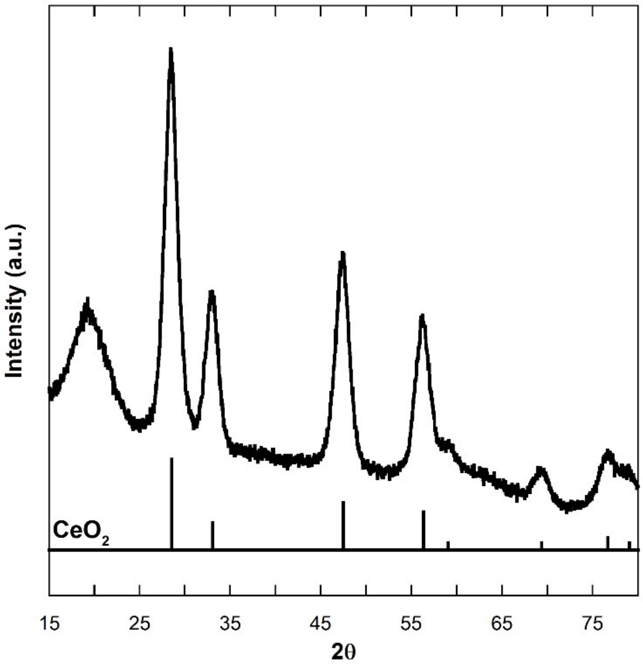

Fig. S2 XRD pattern obtained depositing undoped CeO_2 nanocubes as dried powder. The broad peak around 20° is due to the oleic acid.

The typical XRD pattern obtained for all the samples when deposited as dried powders is shown in Figure S2. All the peaks due to the CeO_2 fluorite structure appear with intensity ratios expected in absence of preferential orientations. A broad peak due to the capping agent is also evident with no sign of any additional impurities and/or other crystalline phases.

Spherical Aberration-Corrected Scanning Transmission Electron Microscopy - Energy Dispersive X-ray Spectroscopy (AC-STEM-EDS)

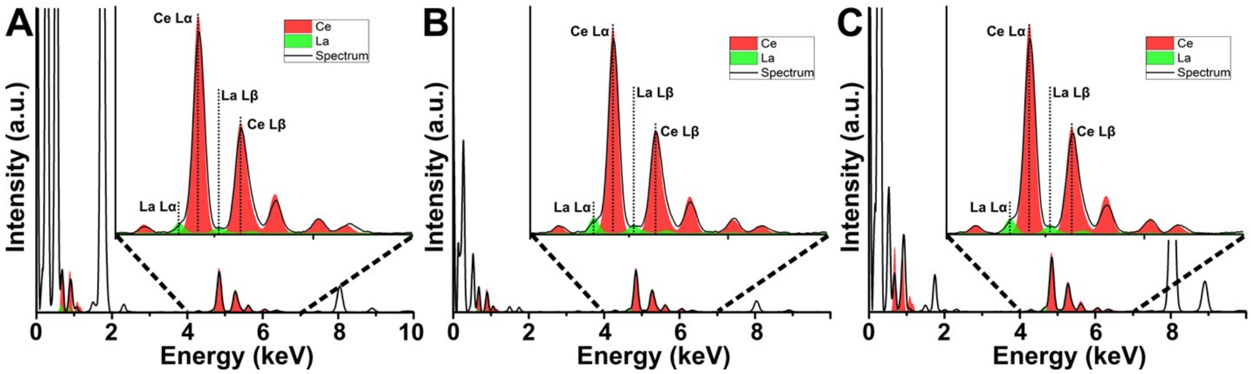

Fig. S3 AC-STEM-EDS full spectra and zoomed areas showing the Lα peaks of Ce and La that were used for the quantification. The deconvoluted Lα peaks of Ce and La are depicted in red and green, respectively. (A) Spectrum corresponding to the elemental maps of the nominal 5 mol% La-doped CeO_2 nanocubes presented in Figure 4D, E. (B) Spectrum corresponding to the elemental maps of the nominal 7.5 mol% La-doped CeO_2 nanocubes presented in Figure 5D, E. (C) Spectrum corresponding to the elemental maps of the nominal 10 mol% La-doped CeO_2 nanocubes presented in Figure 6D, E.

Figure S3A-C shows the AC-STEM-EDS spectra corresponding to the elemental maps presented in Figures 4-6. The quantification was performed using the L peaks of Ce (Lα=4.839 keV, Lβ=5.261 keV) and La (Lα=4.650 keV, Lβ=5.041 keV) and the resulting deconvoluted spectra are shown along with the EDS spectra in the zoomed areas of Figure S3A-C.

Further visible peaks can be identified as C (Kα=0.277 keV), O (Kα=0.525 keV), Si (Kα=1.739 keV, Kβ=1.836 keV) and Cu (Kα=8.040 keV, Kβ=8.904 keV) and are due to spurious contributions coming from the TEM grids and the detector.
